# Value of MR histogram analyses for prediction of microvascular invasion of hepatocellular carcinoma

**DOI:** 10.1097/MD.0000000000004034

**Published:** 2016-07-01

**Authors:** Ya-Qin Huang, He-Yue Liang, Zhao-Xia Yang, Ying Ding, Meng-Su Zeng, Sheng-Xiang Rao

**Affiliations:** a Department of Radiology, Zhongshan Hospital, Fudan University, Shanghai, China; b Shanghai Medical Imaging Institute, Shanghai, China; c Department of Radiology, The Ningbo First Hospital, Ningbo, China.

**Keywords:** diffusion-weighted imaging, hepatocellular carcinoma, histogram, magnetic resonance imaging, microvascular invasion

## Abstract

The objective is to explore the value of preoperative magnetic resonance (MR) histogram analyses in predicting microvascular invasion (MVI) of hepatocellular carcinoma (HCC).

Fifty-one patients with histologically confirmed HCC who underwent diffusion-weighted and contrast-enhanced MR imaging were included. Histogram analyses were performed and mean, variance, skewness, kurtosis, 1th, 10th, 50th, 90th, and 99th percentiles were derived. Quantitative histogram parameters were compared between HCCs with and without MVI. Receiver operating characteristics (ROC) analyses were generated to compare the diagnostic performance of tumor size, histogram analyses of apparent diffusion coefficient (ADC) maps, and MR enhancement.

The mean, 1th, 10th, and 50th percentiles of ADC maps, and the mean, variance. 1th, 10th, 50th, 90th, and 99th percentiles of the portal venous phase (PVP) images were significantly different between the groups with and without MVI (*P* <0.05), with area under the ROC curves (AUCs) of 0.66 to 0.74 for ADC and 0.76 to 0.88 for PVP. The largest AUC of PVP (1th percentile) showed significantly higher accuracy compared with that of arterial phase (AP) or tumor size (*P* <0.001).

MR histogram analyses—in particular for 1th percentile for PVP images—held promise for prediction of MVI of HCC.

## 1 Introduction

Liver transplantation and surgical resection are the potentially curative treatment options for patients with hepatocellular carcinoma (HCC).^[1–3]^ Microvascular invasion (MVI) of HCC had been reported to be an independent prognostic factor for tumor recurrence and survival following curative resection or liver transplantation.^[4,5]^ Sumie et al^[4]^ reported that the 5-year disease-specific survival rates for patients with and without MVI after curative resection for HCC were 59.3% and 92.0%, respectively. Liver transplantation was proved to be harmful in patients with resectable HCC with MVI.^[5]^ Noninvasive assessment of the probability of MVI could be beneficial, as preoperative prediction may allow for treatment optimization, for example, selecting appropriate patient for liver allocation due to the severe organ shortage and predicting prognosis etc.

In daily clinical practice, MVI is always identified by pathology from resection or liver specimens after transplant. The value in the clinical decision-making is currently limited. Tumor biopsy is, still, a matter of debate.^[6,7]^ Studies on methods for prediction of MVI in the patients with HCC included assessment of tumor margin by computed tomography (CT)^[8]^ or gadoxetic acid-enhanced MR imaging,^[9,10]^ CT perfusion analysis,^[11]^ and apparent diffusion coefficient (ADC) measurement,^[12,13]^ etc. However, assessment of tumor margin remains subject to interpretation differences. Furthermore, it does not provide a quantifiable measurement. CT perfusion requires a relatively complex image acquisition and postprocessing algorithm and also delivers relatively high radiation dose. ADC measurement provides only information on the mean value, which does not account for the underlying intra-tumoral heterogeneity.

Histogram analysis is a new approach for quantifying tumor heterogeneity using routinely acquired imaging data and refers to a mathematical approach to evaluate gray-level intensity variations within a region of interest (ROI), which may be used to assess intralesional heterogeneity. Chandarana et al^[14]^ reported that MR enhancement histogram analysis could potentially be used to differentiate clear cell from papillary subtype of renal cell cancer with high accuracy. Studies also demonstrated that histogram analysis of ADC maps had potential value for predicting aggressiveness of endometrial cancer,^[15]^ prostate cancer,^[16]^ and bladder cancer.^[17]^ We hypothesized that histogram analyses of MR enhancement images and ADC maps could assess the histogram distribution of perfusion and cellularity of HCC, which might be used to predict MVI for HCCs preoperatively.

Therefore, the purpose of this study was to assess whether there were any differences in histogram analyses of dynamic contrast-enhanced MR images and ADC maps to discriminate between the 2 groups with and without MVI in patients with HCC.

## 2 Methods

### 2.1 Patients

This retrospective study was approved by Institutional Review Board of our hospital and the requirement for informed consent was waived. We retrospectively evaluated 51 consecutive patients who were suspected with HCC and underwent hepatic MRI between February 2011 and August 2011. Inclusion criteria consisted of histopathologically confirmed HCC by hepatectomy or liver transplantation; without any preoperative cancer-related treatments, that is, trans-arterial chemoembolization and radiofrequency ablation; availability of preoperative dynamic contrast-enhanced (DCE) MR imaging and DWI within 10 days before surgery; without macrovascular invasion on MR imaging.

### 2.2 DWI and DCE-MRI

All patients were performed with a 1.5T MR system (Magnetom Avanto; Siemens Medical Solutions, Erlangen, Germany). All images were obtained in the transverse plane with a body phased-array coil anterior and a spine array coil posterior. Three scan trace DWI (*b* = 0.500 s/mm^2^) in the axial plane with a single-shot, echo-planar sequence was performed before DCE-MRI. A total of 20 to 24 slices were obtained during a 15- to 20-second breath-hold by using generalized autocalibrating partially parallel acquisition (GRAPPA) with R factor of 2. ADC maps were calculated for each diffusion study by the standard console software of the system. The parameters were as follows: 2600/66 (repetition time msec/echo time msec), 128 × 112 matrix, 380–400 × 300–324-mm field of view, 7-cm section thickness with 2.1-mm gap, and 1500 Hz/pixel bandwidth. A 3D T_1_-weighted gradient echo sequence (volumetric interpolated breath-hold examination [VIBE]) with fat-suppression technique was performed before and after the administration of the contrast media. The following parameters were used: 5.04/2.31 (repetition time msec/echo time msec), 12° flip angle, 256 × 192 matrix, 380–400 × 300–324-mm field of view, 24-cm slab thickness resulting in an interpolated 4-mm section thickness, and 300 Hz/pixel/bandwidth. A parallel imaging technique (R factor of 2) was performed with GRAPPA. For DCE-MRI, gadopentate dimeglumine (Magnevist; Bayer Schering Pharma AG, Berlin, Germany, 0.1 mmol/kg) was rapidly administered manually (at a rate of approximately 2 mL/s) by 1 investigator through a 20-gauge intravenous catheter placed in a cubital or cephalic vein. Immediately afterward, a 20-mL saline flush was administered at the same injection rate. Contrast-enhanced images were obtained 20 to 30 seconds for arterial phase (AP), 70 to 80 seconds for portal venous phase (PVP), and 180 seconds for equilibrium phase after the injection.

### 2.3 Histogram analyses

**Figure 1:**
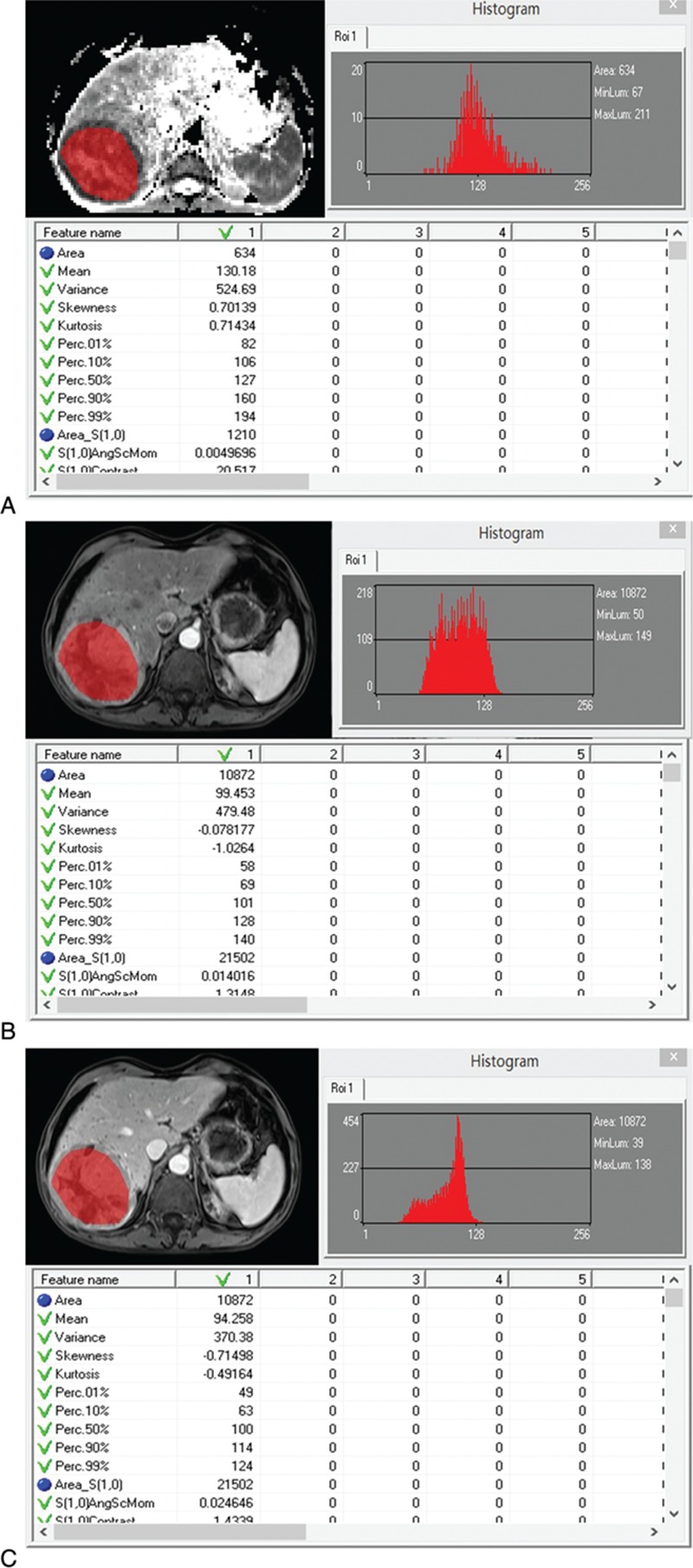
Representative example of regions of interest drawn over tumor on apparent diffusion coefficient (ADC, A), arterial phase (AP, B), and portal venous phase (PVP, C) images. The histogram curves are shown on the upper right. The corresponding histograms (on the bottom) were generated for the ROI and the following parameters were calculated: mean, variance, skewness, kurtosis, and 1th and 10th and 50th and 90th and 99th percentiles.

Histogram analyses were performed by using Mazda (MaZda for Windows, B11 ver. 3.3, www.eletel.p.lodz.pl/programy/mazda/).^[18,19]^ Images were transferred from the hospital's PACS system to an offline workstation. All measurements were performed on the ADC maps, AP and PVP images. Measurements were performed by a single radiologist with 2 years specific experience in abdominal imaging (YQH). For each patient, the radiologist manually delineated free hand ROI around the largest cross-sectional area of the tumor. The largest available tumor was selected as target lesion in case of multiple tumors. Gray-level normalization of each ROI was performed, using the limitation of dynamics to μ ± 3σ (μ, gray-level mean; and σ, gray-level standard deviation), to minimize the influence of contrast and brightness variation. Then histogram data was generated for the ROI (Fig. 1) and the following parameters were calculated: mean, variance, skewness, kurtosis, and 1th and 10th and 50th and 90th and 99th percentiles. These parameters are defined mathematically below. Mean:  
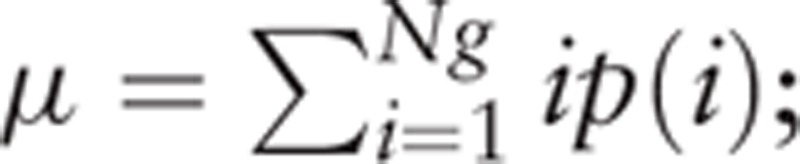
; variance:  
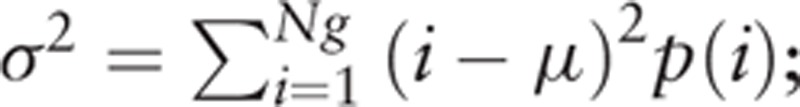
; skewness:  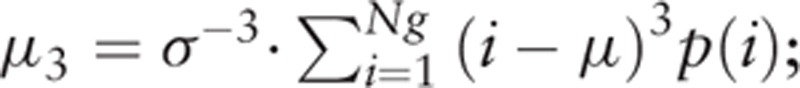; kurtosis:  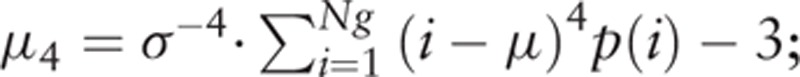; nth percentile is the point at which n% of the voxel values that form the histogram are found to the left. In the formulas, *p*(*i*) is a normalized histogram vector (i.e., histogram whose entries are divided by the total number of pixels in ROI), *i* = 1, 2, …, Ng, and Ng denotes the number of intensity levels.

### 2.4 Standard of reference

Histopathologic evaluation of the surgical resection specimens served as the standard of reference. All specimens were examined by a dedicated pathologist. MVI was defined as the presence of tumor emboli in a portal vein radicle, large capsule vessel, or vascular space lined by endothelial cells only on microscopy.^[20]^ The number of HCCs, histological differentiation, and MVI were recorded. The histopathological grades of the tumors were defined as well differentiated tumors corresponding to Edmondson grade I, moderately differentiated tumors corresponding to Edmondson grade II, and poorly differentiated tumors corresponding to Edmondson grade III or IV.

### 2.5 Statistical analyses

Baseline characteristics of the patients were expressed as mean and standard deviation (SD) or count and proportion. Continuous variables were compared with Student *t* test (or Mann–Whitney *U* test when not normally distributed), and categorical variables were compared by the *χ*^2^ test or Fisher exact test as appropriate. Histogram parameters were compared between the group with MVI from that without MVI using Student *t* test or Mann–Whitney *U* test when not normally distributed. Receiver operating characteristics (ROC) analyses were further constructed to determine the potential diagnostic performance for detecting the presence of MVI. Corresponding areas under the ROC curve (AUCs) with 95% confidence intervals (95% CI) were calculated. Differences in AUCs were analyzed by comparing the ROC curves according to the method of DeLong et al.^[21]^ Statistical analyses were performed using the Statistical Package for the Social Sciences (SPSS, version 16.0, SPSS Inc, Chicago, IL) and MedCalc (MedCalc for Windows, version11.5.0.0, www.medcalc.be). Differences with a *P* value less than 0.05 were considered statistically significant.

## 3 Results

### 3.1 Patient characteristics

The histopathological results revealed that 26 HCCs were positive for MVI, whereas 25 lesions were negative for MVI. The clinical characteristics and histopathological findings in patients with and without MVI are presented in Table 1. Tumor size ranging in largest lesion diameter from 12.1 to 132.2 mm (MVI-positive group: 54.0 ± 37.3 mm; MVI-negative group: 36.0 ± 22.2 mm; *P* = 0.042) showed statistically significant associations with MVI with an AUC of 0.61 (95 CI: 0.46–0.74). For age, sex and a-fetoprotein (AFP), there were no statistically significant differences between the groups with and without MVI. All patients were classified as Child–Pugh A. The poorly differentiated HCCs had high probability of MVI compared with moderately differentiated HCCs (*P* = 0.01).

**Table 1:**
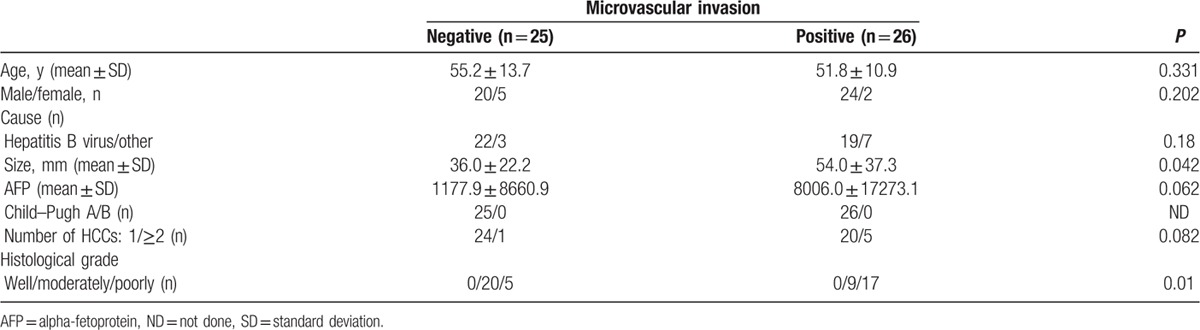
Baseline characteristics of patients with hepatocellular carcinoma (HCC).

### 3.2 ADC map histogram measurements

**Figure 2:**
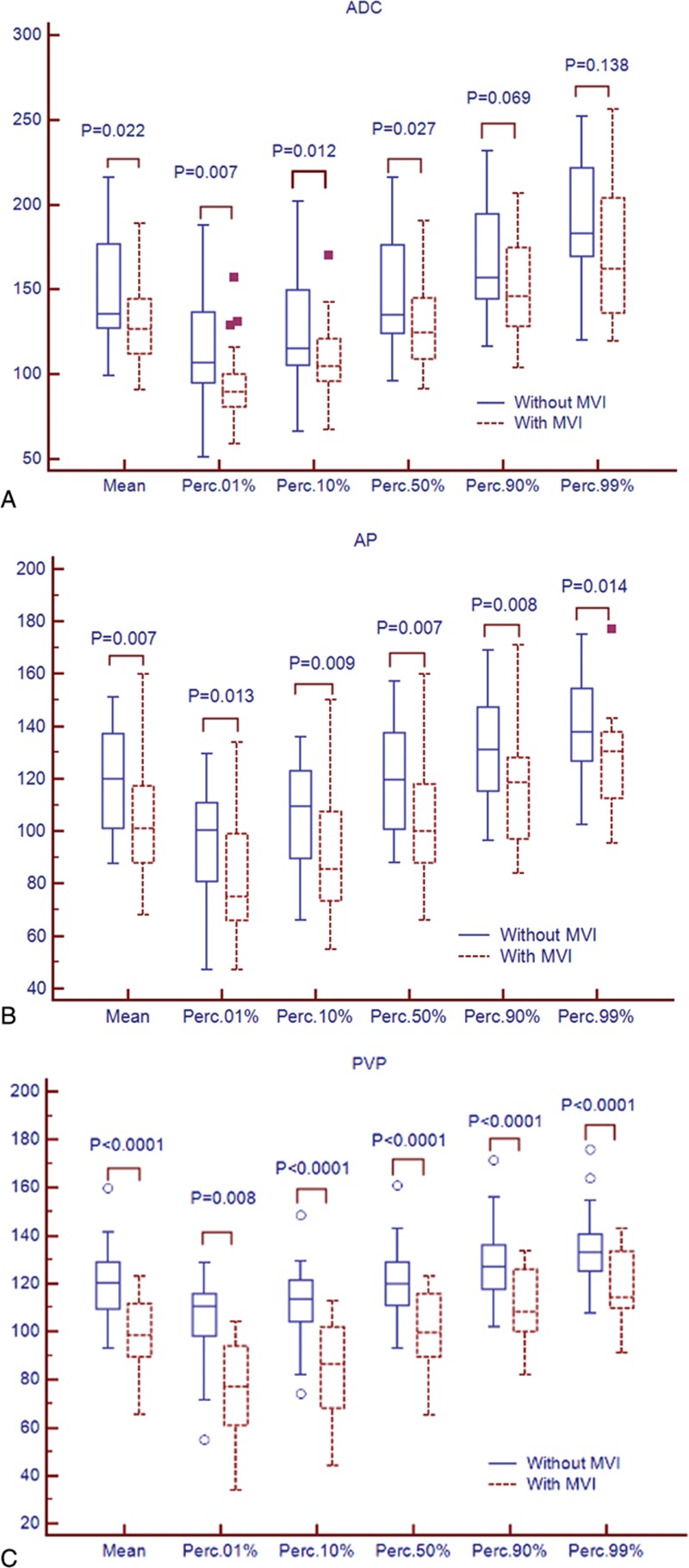
Box-and-whisker plots showed median and interquartile ranges for the histogram parameters for apparent diffusion coefficient (ADC, A), arterial phase (AP, B), and portal venous phase (PVP, C) between hepatocellular carcinoma (HCC) with microvascular invasion (MVI, solid line) and without MVI (dotted line). Points more than one and a half times the interquartile ranges from the median were plotted as outliers.

ADC map histogram measurements are provided in Table 2. Mean, 1th percentile, 10th percentile, 50th percentile of the ADC maps in the patients with MVI were significantly lower than that in the patients without MVI (*P* = 0.007–0.027, Fig. 2A). The AUCs of the above-mentioned significant parameters in prediction of MVI of HCC were 0.66, 0.74, 0.68, 0.66 for mean, 1th percentile, 10th percentile, 50th percentile, respectively. Variance, skewness, 90th, 99th percentile of ADC map showed no significant differences (*P* >0.05).

**Table 2:**
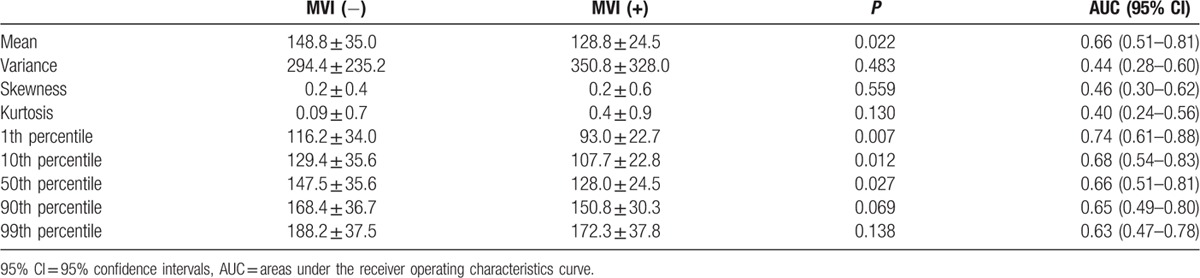
Differences in histogram analyses on apparent diffusion coefficient (ADC) maps between the group with microvascular invasion (MVI) from that without MVI.

### 3.3 MR enhancement measurements

The histogram parameters of AP and PVP for the HCC patients with and without MVI are presented in Table 3. For AP, mean, 1th percentile, 10th percentile, 50th percentile, 90th percentile, 99th percentile showed significant differences between the patients with and without MVI groups (*P* = 0.007–0.014, Fig. 2B), with AUCs of 0.68 to 0.72. There were no differences for variance, skewness, kurtosis (*P* >0.05) for AP. For PVP, mean, variance, 1th percentile, 10th percentile, 50th percentile, 90th percentile, 99th percentile in the patients with MVI were significantly lower than that in the patients without MVI (*P* <0.01, Fig. 2C). The AUCs for detecting the presence of MVI in HCC were 0.83, 0.87, 0.84, 0.82, 0.79, 0.75 for mean, 1th percentile, 10th percentile, 50th percentile, 90th percentile, 99th percentile, respectively.

**Table 3:**
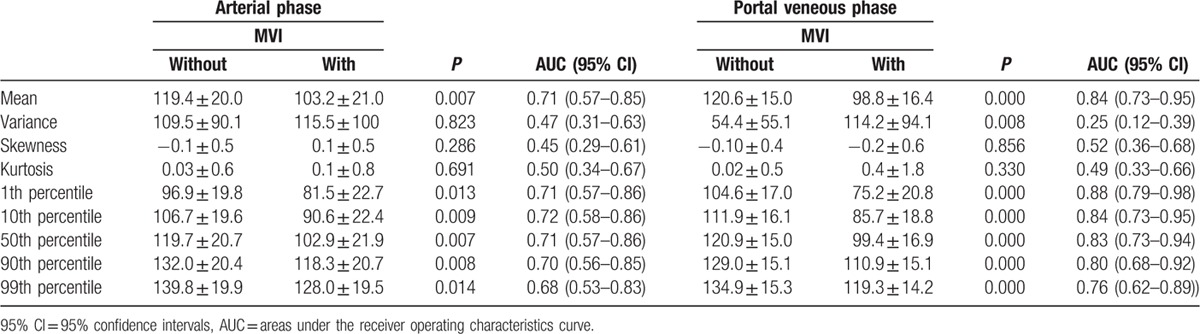
Differences in histogram analyses on arterial phase (AP) and portal veneous phase (PVP) images between the group with microvascular invasion (MVI) from that without MVI.

### 3.4 Comparison of ROC curves

**Figure 3:**
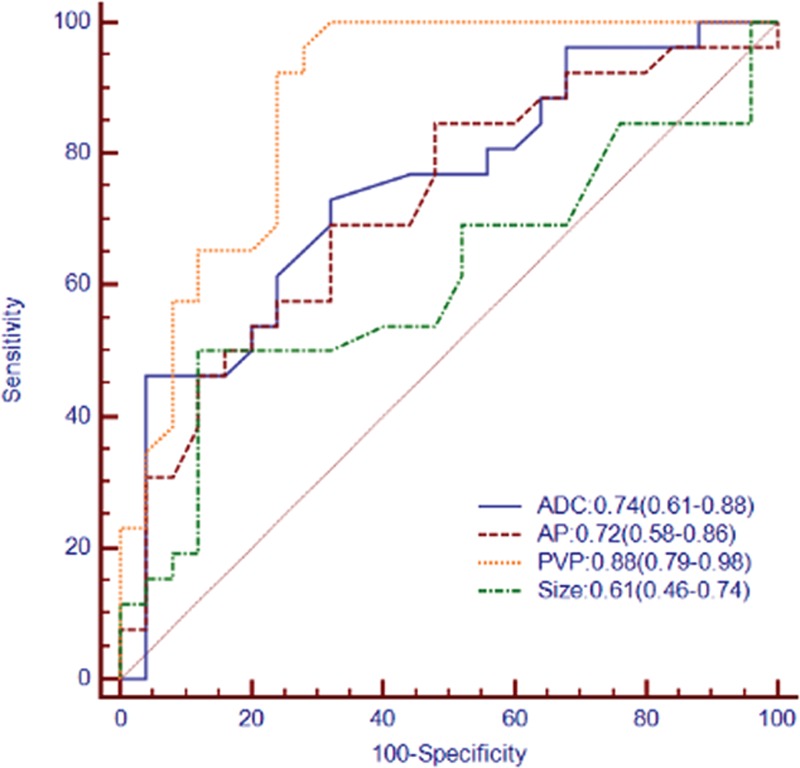
Receiver operating characteristic (ROC) and areas under the curve (AUC) for prediction of microvascular invasion (MVI) in hepatocellular carcinoma by histogram analyses for apparent diffusion coefficient (ADC) maps, arterial phase (AP), portal venous phase (PVP) images, and tumor size. Numbers in parentheses represent 95% confidence intervals.

Tumor size, the highest AUCs of histogram parameters for ADC maps (1th percentile), AP (10th percentile), PVP images (1th percentile) were used to compare the diagnostic performance of presence of MVI in HCC (Table 4). Comparison of ROC curves were displayed in Fig. 3. For the histogram analyses, the largest AUCs were 0.74 (1th percentile) for ADC maps, 0.72 (10th percentile) for AP, and 0.88 (1th percentile) for PVP images. Histogram analyses of PVP images were significantly higher accuracy compared with that of AP images or tumor size (*P* <0.001). The differences in AUCs among histogram analyses of ADC maps, AP images, or tumor size were not significant (*P* = 0.07–0.80).

**Table 4:**
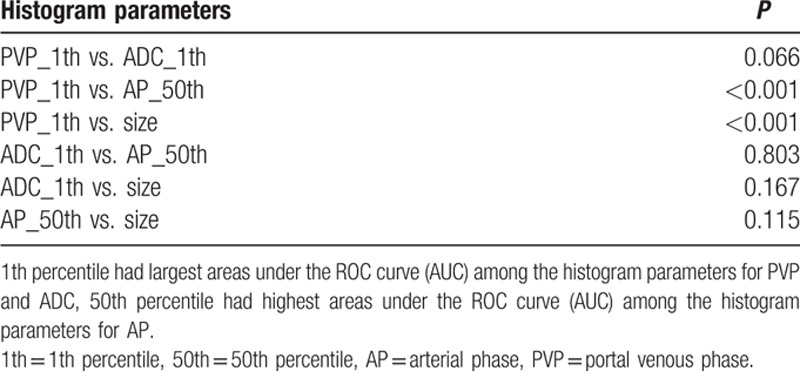
Comparison of receiver operating characteristics (ROC) curves of histogram analyses for apparent diffusion coefficient (ADC) maps, contrast enhancement images, and tumor size for presence of microvascular invasion (MVI).

## 4 Discussion

The goal of our study was to evaluate the value of MR histogram analyses for preoperatively predicting the presence of MVI of HCC. The results of our study indicated that the histogram-derived parameters for PVP images provided high accuracy for identification of MVI of HCC with AUCs up to 0.88 and showed significantly better diagnostic performance compared with that of ADC maps, AP images, or tumor size according to ROC analyses. ADC histogram analyses resulted in AUCs up to 0.74. AP images histogram analyses (AUC: 0.45–0.72) and tumor size (AUC: 0.61) showed low accuracy for predicting MVI. The diagnostic performance for histogram-derived 1th percentile of PVP images and ADC maps increased compared with mean values (AUC: 0.88 vs. 0.74 for 1th percentile; 0.84 vs. 0.66 for mean values).

MVI is defined as the presence of tumor emboli in a portal vein radicle, large capsule vessel, or vascular space lined by endothelial cells only on microscopy,^[20]^ which is at level beyond the resolution of routine imaging data. In theory, MVI could change perfusion of the tumor by invading the branch of portal venous. And the poorly perfusion is associated with higher tumor grades, poorer treatment response, higher probability of metastatic disease, and shorter progression-free survival.^[22,23]^ Wu et al^[11]^ demonstrated that portal vein flow calculated from liver CT perfusion in HCC with MVI was significantly higher compared with HCC without MVI, but there was no significant difference for hepatic artery flow. Our study showed the similar results that PVP images histogram analyses outperformed AP images. The possible explanations might be that MVI mainly affected portal vein perfusion of tumor, besides tumor heterogeneity is determined by heterogeneous vascularization, cell density, necrosis, and fibrosis of a tumor.^[24]^ So the differences in contrast-enhancement of HCC between with and without MVI were more evident for PVP images than AP images. Previous studies reported that tumor size was supposed to be critical factors for prediction of MVI in HCC.^[25,26]^ However, some studies demonstrated that tumor size was not an independent predictor of MVI by multivariate logistic regression analysis.^[9,27]^ We found that tumor size showed less accuracy for detection of MVI in comparison with histogram analyses of PVP images.

DWI is a functional MR imaging technique that uses differences in the extracellular movement of water molecules to discriminate between tissues of varying cellularity. ADC can provide quantitative information related to the tissue cellularity and integrity of cellular membranes, as well as microcapillary perfusion by reflecting the molecular diffusion of water and perfusion.^[28,29]^ Previous studies demonstrated that lower ADC values can be a useful predictor of MVI.^[12,13]^ However, the AUCs of ADC histogram parameters for predicting MVI in our results were not very high (up to 0.73). The possible explanations might be that absolute ADC values are dependent on technical parameters and have been shown to vary between vendors;^[30]^ lower imaging resolution of ADC maps (matrix: 128 × 112 in our study), which might be insufficient to reveal histogram distribution features of the voxels; DWI was performed by using only 2 *b* values (0 and 500 seconds/mm^2^), which might affect the accuracy of ADC calculation. Theoretically, ADC measurement with DWI obtained with multiple *b* values could reduce the measurement error.

As compared with mean values of PVP images and ADC maps measurements, 1th percentile had higher diagnostic performance for prediction of MVI (AUC: 0.88 vs. 0.74 for 1th percentile; 0.84 vs. 0.66 for mean values), which might be explained by different heterogenous biologic and perfusion characteristics between HCCs with and without MVI. Our findings showed that the 1th percentile PVP images and 1th percentile ADC maps for HCC with MVI showed significantly lower than that of HCC without MVI. The reason may be that HCCs with MVI have higher cellularity and/or decreased capillary perfusion.^[13]^ Our results suggested that heterogeneity of capillary perfusion and/or cellularity of HCCs were represented to a great extent by 1th percentile of PVP images and ADC maps than by mean values. This was in keeping with the results of previous study. Donati et al^[16]^ reported that 10th percentile ADC showed significantly higher accuracy than mean ADC in differentiating low-grade from intermediate- or high-grade prostate cancer.

There are several limitations in our study. First, because of its retrospective nature, the possibility of a selection bias cannot be excluded; second, we performed DWI using only 2 *b* values of 0 and 500 seconds/mm^2^. However, our results showed histogram of ADC maps had the potential value for prediction of MVI in HCC, despite the relative low AUC (up to 0.73). Recently, studies showed that intravoxel incoherent motion DW-MR imaging applying multiple *b* values might be a promising tool to improve data reproducibility^[31]^ and assess histopathological features of HCC.^[32]^ Histogram of intravoxel incoherent motion (IVIM) might hold the promise for detecting MVI.

In conclusion, histogram analysis of PVP images showed higher accuracy compared with that of ADC, AP, or tumor size for discriminating HCCs with MVI from HCCs without MVI. Moreover, the 1th percentile of PVP images might be the best parameter for risk stratification in patients with HCC at the time of diagnosis. Because of limited sample size, we did not go further on proposing and validating an algorithm with the clinical, laboratorial, and MR histogram markers. The current results are obviously still premature and warrant further validation of these preliminary data presented by large and prospective patient studies.
